# Recent advances and current challenges in population genomics of structural variation in animals and plants

**DOI:** 10.3389/fgene.2022.1060898

**Published:** 2022-11-29

**Authors:** Ivan Pokrovac, Željka Pezer

**Affiliations:** Laboratory for Evolutionary Genetics, Division of Molecular Biology, Ruđer Bošković Institute, Zagreb, Croatia

**Keywords:** genomic structural variation, structural variants, copy number variation, population genomics, evolution, adaptation, speciation

## Abstract

The field of population genomics has seen a surge of studies on genomic structural variation over the past two decades. These studies witnessed that structural variation is taxonomically ubiquitous and represent a dominant form of genetic variation within species. Recent advances in technology, especially the development of long-read sequencing platforms, have enabled the discovery of structural variants (SVs) in previously inaccessible genomic regions which unlocked additional structural variation for population studies and revealed that more SVs contribute to evolution than previously perceived. An increasing number of studies suggest that SVs of all types and sizes may have a large effect on phenotype and consequently major impact on rapid adaptation, population divergence, and speciation. However, the functional effect of the vast majority of SVs is unknown and the field generally lacks evidence on the phenotypic consequences of most SVs that are suggested to have adaptive potential. Non-human genomes are heavily under-represented in population-scale studies of SVs. We argue that more research on other species is needed to objectively estimate the contribution of SVs to evolution. We discuss technical challenges associated with SV detection and outline the most recent advances towards more representative reference genomes, which opens a new era in population-scale studies of structural variation.

## Changing definition and typical properties

Genetic variation is the major focus of population genetics as it provides raw material upon which evolutionary forces act to create phenotypic diversity. Over the past two decades, it has become evident that variation in the linear structure of the genome is taxonomically ubiquitous and that it affects a much larger portion of the genome than the variation in the sequence itself (Huddleston et al., 2017; Kosugi et al., 2019; Hämälä et al., 2021; Box 1). This form of genetic variation results in structural variants (SVs) that can affect orientation (inversions), position (translocations), or copy number. The latter are collectively termed copy number variants (CNVs) and include deletions, insertions, and amplifications of a sequence. A specific group of CNVs termed presence-absence variations (PAVs) refers to sequences that exist in some genomes while completely missing in other genomes of the same species (Saxena et al., 2014). SVs were first defined as events of at least 1 kilobase pairs (kbp) in length (Feuk et al., 2006) but the definition has since expanded to encompass sizes down to 50 bp and larger (Alkan et al., 2011; Sudmant et al., 2015b). Our increasing understanding of the prevalence of SVs as major contributors to genetic variation has led to the inclusion of other genome rearrangements and elements in this definition, that were long before known to have a variable structure within population. The current definition based on SV size also includes interspersed elements (such as transposable elements; TEs), tandem repeats (including micro-, mini-, and macrosatellites) as well as aneusomy and aneuploidy (Pös et al., 2021).

Spontaneous, *de novo* SVs occur several hundred-fold less frequently than point mutations (Belyeu et al., 2021), although the mutation rate varies considerably by SV type (Collins et al., 2020). Recent large family-trio studies in humans and rhesus monkeys estimated that less than one *de novo* CNV is formed per genome per generation (Belyeu et al., 2021; Thomas et al., 2021). Interestingly, parental age does not affect the rate of these mutations in either species, in contrast to single nucleotide variants (SNVs), which accumulate with paternal age in both species (Kong et al., 2012; Wang et al., 2020). This difference between SNVs and CNVs was proposed to be due to the mechanism of their formation—CNVs are thought to form during meiosis which occurs only once per generation, whereas SNVs can arise as errors during replication in mitosis or unrepaired DNA damage—processes which occur frequently over a lifetime in the germline (Thomas et al., 2021).

Some genomic regions show an extraordinary propensity for structural variation such that they reach mutation rates hundreds and thousands of times higher than nucleotide substitutions, according to some estimates (Zhang et al., 2009). These are referred to as recurrent SVs. Their high mutability is attributable to the repetitive architecture of the genomic region in which they reside, which enables non-allelic homologous recombination (NAHR). Among all known mechanisms of SV formation, NAHR is thought to occur the most frequently, when two highly similar but non-allelic DNA sequence repeats align and crossover during meiosis, causing deletion, duplication, or inversion of the region between the repeats, depending on the orientation of the aligned sequences (Zhang et al., 2009). These mediators of NAHR are usually considered to be CNVs themselves as they exist in the genome in variable low or high copy numbers, such as segmental duplications, transposable elements, and tandem repeats. Other mechanisms of SV formation such as non-homologous end joining (NHEJ), microhomology-mediated break-induced replication (MMBIR), fork stalling and template switching (FoSTeS), and replication slippage are not dependent on high sequence similarity and create mainly non-recurrent SVs. These mechanisms and events are usually discussed in the context of genomic disorders (Hastings et al., 2009; Carvalho and Lupski, 2016), although they may contribute to natural polymorphism without seemingly negative effects.

## Effect on gene expression and phenotypic variation

The high mutability of SVs is reflected in their high variability within population. For example, it is currently estimated that any human individual contains on average 16 Mb of structural variation (Ebert et al., 2021) or up to 27,000 SVs, including highly repetitive elements (Chaisson et al., 2019; see Box 1). According to the data from NCBI’s database of human genomic structural variation (dbVar), almost 100,000 regions in the human genome are affected by SVs at population frequency ≥1% (Box 1). Given this abundance and high variability within population, SVs are expected to have a large impact on phenotypic variation. However, determining the functional effects of the majority of SVs is difficult, especially in natural populations which are not readily amenable to genetic manipulations (Lauer and Gresham 2019). The association of SVs with gene expression remains the most commonly used proxy for assigning phenotypic consequences. An ever-increasing number of population-scale studies have emerged to suggest that SVs of all types contribute to phenotypic variation on multiple layers of gene regulation. CNVs can alter gene dosage (Handsaker et al., 2015) and thus directly affect protein levels, as shown for the human salivary amylase gene (Perry et al., 2007). Structural variants can also modulate gene expression by re-organizing chromatin domains. Perturbations of topologically associated domains (TADs) can lead to the formation of novel regulatory modules, as shown in humans, apes, and mice (Spielmann et al., 2018; Fudenberg and Pollard, 2019; Gilbertson et al., 2022). CNVs can encompass regulatory elements, such as in the case of an enhancer that controls a gene *NDP* that is responsible for wing pigmentation in pigeons (Vickrey et al., 2018). Expression of this gene is positively correlated with both increased melanism and enhancer copy number. In crows, the same gene is associated with plumage variation but is controlled by a different SV type - an LTR retrotransposon insertion that causes reduced expression (Weissensteiner et al., 2020). SVs can affect whole regulatory networks by affecting single key transcription factors and thus have a large phenotypic effect. This was recently exemplified by a mutation in the *ENO* gene, which encodes a transcription factor that regulates floral meristem size in tomatoes - an 85-bp deletion in the promoter of *ENO* was shown to be responsible for the increase in fruit size during tomato domestication (Yuste-Lisbona et al., 2020). Copy number variation in introns causes variable gene length and is commonly found in healthy human populations. These CNVs reside inside genes with essential functions and are proposed to be responsible for their differential regulation between individuals (Rigau et al., 2019). A recent genome-wide association study (GWAS) based on presence-absence variations in rapeseed identified PAVs among different ecotypes that altered the expression of genes responsible for flowering regulation (Song et al., 2020).

While these and other studies illustrate the contribution of individual SVs to phenotypic variation *via* gene regulation, they do not attest to the extent to which SVs explain overall variation in gene transcription within population. Several studies to date have attempted to ascertain the causality of SVs at expression quantitative trait loci (eQTLs). The most comprehensive study thus far, performed in humans and based on over 600 individuals and 48 tissues, found that SVs are causal at 2.66% of eQTLs which represents a tenfold enrichment relative to their abundance in the genome (Scott et al., 2021). This study revealed that, among all SV types, multiallelic CNVs, both coding and non-coding, have the highest association with eQTLs and that the contribution of transposable element insertions was small. Prior estimates based on a limited number of samples and tissues are in discordance with the study by Scott et al. (2021), as they found either a much larger or much smaller proportion of eQTLs to be caused by SVs. For example, a study based on 13 tissues from 147 individuals estimated up to 6.8% of eQTLs are driven by a causal SV (Chiang et al., 2017). An earlier study associated only 0.56% of eQTLs with SVs (Sudmant et al., 2015b), but it was based on a single cell line although the number of individuals was comparable to the study performed by Scott et al. (2021). This large disagreement in estimates between studies suggests that future efforts should employ a more exhaustive number of tissue types, and possibly target a variety of biological processes, to more precisely assess the contribution of SVs on gene expression in a tissue- and condition-specific manner. Indeed, genes with tissue-specific expression exhibit greater copy number variability than genes with widespread expression (Dopman and Hartl, 2007; Henrichsen et al., 2009; Keel et al., 2016), suggesting that SVs more often have roles in specialized rather than general processes. A recent study based on only two tissue types in three-spined sticklebacks found a strong positive correlation between gene copy number and expression in almost 40% of analyzed CNVs (Huang et al., 2019). Such high association becomes less surprising when one considers that gene-encompassing CNVs were previously found to be enriched for immune activity genes in sticklebacks and that the study focused on immune tissues where these genes are expected to be expressed. Another study identified thousands of tandemly repeated minisatellite sequences variable in copy number within population to be associated with local expression and DNA methylation levels (Garg et al., 2021). These CNVs were associated with genes that have been linked with human phenotypes through genome-wide association studies and were strongly enriched for regulatory elements such as enhancers and promoters, suggesting that these non-coding multiallelic CNVs may be causal for human phenotypes and have regulatory functions.

In summary, multiallelic CNVs seem to be a class of SVs that is the most strongly implicated in the contribution of SVs to variation in gene expression. However, the presented figures are likely underestimates. We can expect to approach more precise estimates with the addition of a more comprehensive set of tissues and by analyzing diverse biological conditions in future studies. Despite the large discrepancies in estimates, current knowledge collectively suggests that both coding and non-coding SVs may have a tremendous impact on gene expression, and thus affect phenotypes in the ways we are just beginning to understand. GWASs based on SNVs have not been able to completely identify the genetic components underpinning (human) traits and disorders; over the past decade, a growing body of evidence has accumulated to suggest SVs as a source of this “missing heritability” (Sudmant et al., 2015b; De Coster et al., 2021; Garg et al., 2022; Zhou et al., 2022).

## Impact on evolution

Hundreds of CNVs can be found in the genomes of healthy individuals and they show strong signatures of population structure in numerous species (Sudmant et al., 2015a; Pezer et al., 2015; Xu et al., 2016). This has been used as an argument to propose that the majority of CNVs evolve under neutral evolutionary pressures, such that the patterns of copy number variation seen in populations are mainly shaped by demographic events, mutation rate, and genetic drift (Iskow et al., 2012). However, even such generalizations of the evolutionary implications of CNVs (and other SVs) should be considered in their functional contexts. Recent studies in humans and rhesus monkeys revealed that *de novo* gene deletions outnumber duplications by several times (Belyeu et al., 2021; Thomas et al., 2021), but this ratio becomes skewed over time, as illustrated by the proportion of fixed gene losses along the primate lineage, which becomes smaller (Fortna et al., 2004; Dumas et al., 2007; Sudmant et al., 2013; Thomas et al., 2021). This suggests that, over generations, purifying selection acts against deletions of complete genes. The vast majority of SVs seem to be depleted from functional regions of the genome and segregate at low frequencies, as shown by studies in different species (Pezer et al., 2015; Hämälä et al., 2021). Signals of pervasive selection against all types of SVs that overlap genes, except whole-gene duplications, have recently been discovered in a large analysis of thousands of human genomes (Collins et al., 2020). These studies collectively suggest that most SVs affecting genes are deleterious. A somewhat contrasting observation came from a recent study that suggested that SVs significantly contribute to non-neutral variation in humans (Saitou et al., 2022). Assuming that majority of SVs evolve neutrally, this study looked for SVs with unusual allele frequency distribution among populations and came to a surprising number of over 500 putatively adaptive SVs in humans. A proportion of these included SVs that affect exons and were dominated by multiallelic CNVs.

### Contribution to adaptation

Structural variants exist in extremely heterogeneous forms, in terms of type (insertion, deletion, duplication, inversion, and translocation), size, mutation rate, and genomic context. Consequently, even without technical difficulties in their discovery, they constitute a substantial challenge for evolutionary studies. While the current picture of the evolutionary effects of SVs remains incomplete, their contribution to adaptive evolution and diversification is becoming more evident (Radke and Lee, 2015; Saitou et al., 2022). An increasing number of studies suggest that SVs are involved in a variety of adaptations in a range of taxonomic groups, affecting different biological systems such as immunity, metabolism, and sensory perception. Instances of naturally occurring parallel ecological divergence provide an especially useful framework for detecting potentially adaptive SVs. The idea is that if the frequency of an SV is higher in a derived population of a certain ecotype compared to the ancestral population of a different ecotype, and this is observed repeatedly in multiple independent populations, that SV is likely contributing to the adaptive phenotype. Adaptation of marine fish to freshwater represents such a system. A study by Ishikawa et al. (2019) found that a gene involved in fatty acid desaturation was duplicated in freshwater lineages. Transgenic manipulation of this gene enabled marine lineages to produce fatty acids and survive in freshwater that lacks fatty acids. This suggested that differences in gene dosage contribute to differences in survival on fatty acid–deficient diets. In a follow-up study, additional gene duplications were identified to be associated with freshwater colonization, including genes involved in immune function and thyroid hormone metabolism (Ishikawa et al., 2022). In another study, two large chromosome inversions were identified to exhibit parallel association with freshwater adaptation (Zong et al., 2021). These inversions contained multiple genes involved in various processes such as metabolism, immunoregulation, growth, maturation, and osmoregulation, thus potentially affecting morphology, physiology and behavior. It was recently found that large inversions were common and widespread in natural populations of deer mice and several inversions with significant differences in allele frequency between forest and prairie ecotypes were identified, which likely contribute to local adaptation (Harringmeyer and Hoekstra, 2022). It has been proposed that among all SVs, chromosomal inversions are the most frequently linked to adaptive traits (reviewed in Wellenreuther and Bernatchez 2018). However, a wealth of studies suggests that CNVs may be comparable if not even dominant in this aspect. Since the initial discovery of copy number variation, more and more instances of CNVs with a putative role in local adaptation of human populations are emerging (Iskow et al., 2012; Hsieh et al., 2019; Quan et al., 2021; Saitou et al., 2022). Both deletions and duplications are implicated. For example, recurring exonic deletions in the haptoglobin gene were shown to contribute to human health by lowering cholesterol levels in the blood (Boettger et al., 2016). Copy number variations in genes *Ppd-B1* and *Vrn-A1* contribute to global adaptation of wheat to a wide range of environmental conditions (Würschum et al., 2015). These genes modulate the timing of flowering and their increase in copy number is associated with altered expression (Díaz et al., 2012). Furthermore, an increase in *EPSPS* gene copy number confers resistance to the herbicide glyphosate in different weed species (Gaines et al., 2010; Baek et al., 2021). Similarly, triplication of a gene associated with aluminum tolerance in some maize lines correlates with increased expression, which confers higher tolerance to aluminum in maize grown on acidic soils (Maron et al., 2013). Huang et al. (2019) studied the role of gene copy number in adaptation to distinct parasite environments between the lake and river habitats in sticklebacks. In some of these genes, copy number was differentiated between ecotypes and it positively correlated with transcript level, suggesting that gene dosage contributes to local adaptation by modulating expression. Similarly, specific SVs with signs of local adaptation were recently uncovered in chocolate tree, some of which are linked to genes that are also differentially expressed between populations (Hämälä et al., 2021). They were enriched for functions related to immunity, emphasizing the role of SVs in local adaptation to specific pathogens. In the fruit fly, hundreds of TEs were identified to be associated with expression variation of nearby genes, some of them bearing adaptive signatures (Rech et al., 2022). Gene loss can also produce adaptive phenotypes, as suggested for polar bear evolution, where a considerable number of genes encoding olfactory receptors have been lost, as well as the salivary amylase-encoding gene and genes involved in fatty acid metabolism (Rinker et al., 2019). These CNVs evolved rapidly over a short evolutionary period, driven by a dietary shift from omnivorous to carnivorous during polar bear evolution. Even some gene retrocopies show signatures of positive selection, as shown by recent studies in humans and mice (Schrider et al., 2013; Zhang and Tautz 2022).

### Contribution to speciation

Changes in genome structure can lead to incompatibilities between populations and thus enhance speciation. SVs can enable reproductive isolation through various mechanisms such as suppressed recombination, hybrid incompatibility, and intrinsic postzygotic or premating isolation (reviewed in Zhang et al., 2021). Inversions, especially large ones, seem to be particularly implicated in suppressing recombination. In heterozygotes for such SV, inverted region fails to pair with non-inverted allele during meiosis, preventing them from cross-over. This results in both variants independently accumulating mutations in their sequences over time, creating “genomic islands of divergence,” which eventually leads to incompatibilities (Zong et al., 2021). Incompatibility can also be caused by CNVs, especially if affecting the whole gene, as exemplified by a duplication of a key photosynthetic gene in the yellow monkeyflower (Zuellig and Sweigart, 2018). This variant causes lethality in naturally occurring hybrids between two closely related species, presumably by misregulated transcription. Copy number variation can also play a role in assortative mate choice, as suggested for hundreds of CNVs found to be associated with reinforcement of sexual isolation between the two European subspecies of the house mouse (North et al., 2020). Premating isolation can even be mediated by TE, as shown for the 2.25-kb LTR retrotransposon insertion which affects plumage in birds, a trait associated with prezygotic isolation through social and sexual selection (Weissensteiner et al., 2020). This study nicely illustrates how even a single and small SV can change the evolutionary trajectory of a population and potentially lead to divergence and speciation. Translocations represent a special type of SVs, in that they are often associated with genome instability and negative outcome such as infertility and oncogenesis (Aplan, 2006; Mitelman et al., 2007). Rare instances are found as naturally occurring polymorphisms in healthy individuals. One of the well-studied examples is Robertsonian fusion in the house mouse subspecies *Mus musculus domesticus*, which refers to the translocation of the whole chromosome arm, *i.e.* the joining of two telocentric chromosomes to create a metacentric chromosome. Robertsonian fusions are more frequent in small and geographically isolated populations and they are proposed to contribute to reproductive isolation (Garagna et al., 2014). Similar to inversions, translocations have been associated with the suppression of recombination and have recently been implicated in genetic divergence between subspecies of bananas (Martin et al., 2020) and populations of spiny frogs (Xia et al., 2020).

### Structural variants as loci of large effect

Some SVs are large enough to span many genes and regulatory regions. Consequently, they can simultaneously affect multiple traits, acting as supergenes of large effect. Such a role has often been assigned to large inversions, proposed to be associated with complex phenotypes (reviewed in Wellenreuther and Bernatchez 2018). An inversion that contains multiple advantageous alleles will be more strongly selected for than an inversion containing a single favorable gene variant. These alleles are also more likely to be coinherited due to suppressed recombination in heterokaryotypes, contributing further to their rapid increase in frequency in the population under selection. Consequently, large inversions are considered to have significant roles in rapid environmental adaptation and speciation. SVs can also cause dramatic changes in the regulation of multiple genes by disrupting chromatin domains and exposing certain promoters to certain enhancers for the first time. It was proposed that translocations and inversions perturbed TADs and thus created differences in promoter-enhancer connections between humans and mice that are responsible for differential regulation of genes involved in immune response between the two species (Gilbertson et al., 2022). SVs are otherwise strongly depleted from TAD boundaries and active chromatin states, suggesting that they are under negative selection (Fudenberg and Pollard, 2019). Single SVs often impact the expression of multiple genes, two on average in humans (Scott et al., 2021), suggesting that they frequently exert a pleiotropic effect on phenotypic diversity. Evidence of an SV with a strong and immediate effect on phenotype came from a recent experimental evolution study on nematode. Zhao et al. (2020) studied the genetic basis of adaptation to food sources in *Caenorhabditis elegans* and found a recombinant inbred line with increased fitness. They detected a complex SV as its genetic basis; this complex rearrangement caused duplication of a gene involved in exploration behavior and modified its expression. It was proposed that the SV occurred as a single genomic instability event and became fixed in a population because it provided a fitness advantage in a new environment. These findings highlight the potential of SVs in causing dramatic structural changes in the genome which can substantially and instantaneously affect phenotypes. The majority of such large events are expected to be deleterious. However, under specific circumstances, some variants may provide a strong selective advantage which would enable them to quickly rise in frequency within the population and even become fixed over a short evolutionary time.

## Population-scale studies of SVs are strongly biased toward humans

**FIGURE 1:**
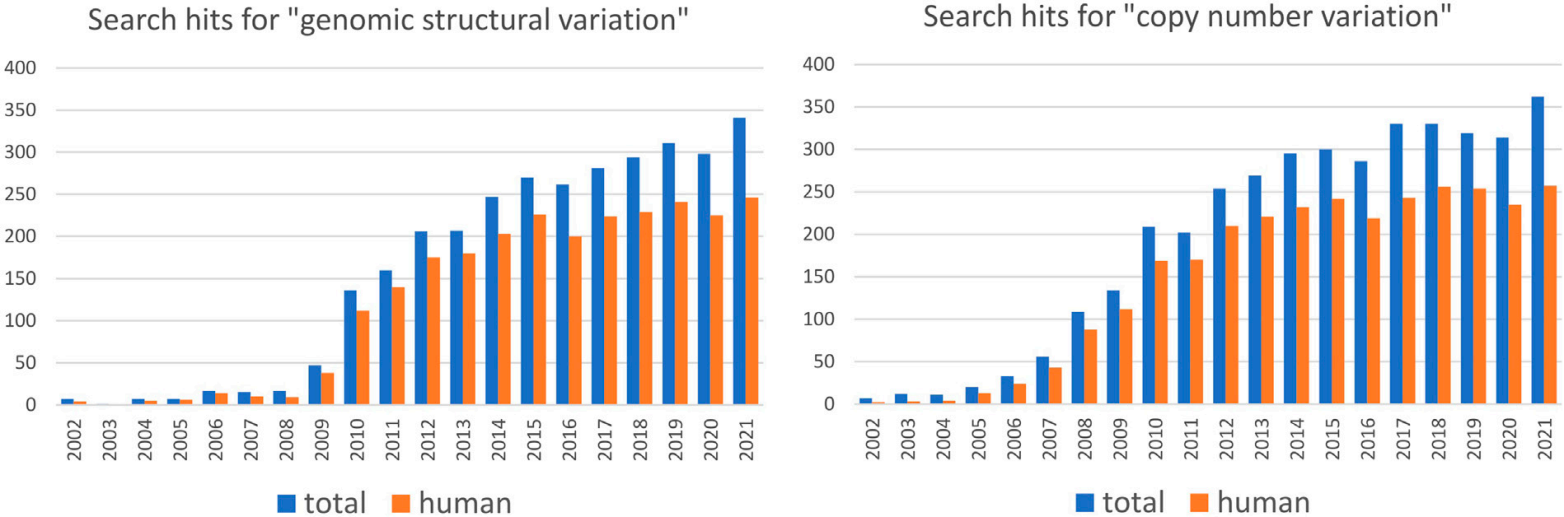
Number of articles per year containing indicated search term in PubMed in the period from 2002 to 2021. Search is performed for the combination of indicated term AND “population” (data in blue) or for indicated term AND “population” AND “human” (data in red). The data suggest that the majority of population-scale SV and CNV studies are based on humans.

Studying genetic variation in natural populations is crucial for understanding how genomes evolve. Assessing the degree of structural variation in various species and populations contributes to our general understanding of its role in evolutionary processes. Nevertheless, population-scale studies of SVs are heavily biased toward humans (Figure 1) and insights gained mainly from studies on human populations guide our general perception of structural variation (Box 1). However, modern humans have a specific population history that involved at least one severe bottleneck followed by rapid expansion and repeated founder effect (Watkins et al., 2001; Amos and Hoffman, 2010), which resulted in substantially lower genetic diversity compared to many other species. Moreover, genetic boundaries between human populations are often blurry, reflecting the frequent population movement and admixture. Humans are also characterized by a small effective population size (Tenesa et al., 2007; Park 2011), which is known to reduce the efficacy of natural selection and increase the influence of genetic drift. Thus, human populations by no means embody a “typical” evolutionary trajectory and more studies of SVs in non-human populations are needed to disentangle the roles that SVs play in evolution and ecological specialization. Based on growing evidence, SVs may be the key players of rapid adaptation to changing environments and naturally occurring examples of parallel evolution represent excellent opportunities to study the genetic architecture of rapid adaptation, such as adaptation to freshwater discussed above. The independence of studied populations that converged adaptive traits is desirable: the stronger the evidence that they independently evolved similar traits under the same selective pressure, the stronger the association with the underlying variant. Population studies in non-human species may provide more instances of such independent, parallel evolution as a framework for studying the role of SVs in adaptation and speciation. For instance, adaptation to the subterranean environment has been documented for many taxa, yet the impact of structural variation in this context is still unexplored. Numerous examples of parallel evolution can also be found in domesticated species, and evidence of SVs playing a part in trait evolution during domestication in plants and animals is emerging. For example, white coat color was independently selected for in sheep and goats - in both species, this trait is associated with duplication of the agouti signaling protein (*ASIP*) gene (Norris and Whan, 2008; Fontanesi et al., 2009). In plants, the loss of seed shattering was repeatedly selected for during domestication and is often associated with a deletion in gene *Sh1* in different cereal species (Lin et al., 2012; Choi et al., 2019). From an evolutionary point of view, domestication is a very specific process that usually involves a population bottleneck that substantially decreases genetic diversity and increases the frequency of domestication alleles (Gaut et al., 2018). It has been proposed that, at least in plants, deletions underly some of the crucial domestication traits, whereas during later stages of domestication (*i.e.* during diversification) various SV types facilitate local adaptation (Gaut et al., 2018; Lye and Purugganan, 2019). Hence, although they may provide some interesting examples of parallel evolution, domesticated species may not represent a general model for studying the role of SVs in evolution.

## Challenges in the detection of structural variants

There is no doubt that sequencing technology based on short reads has tremendously advanced our knowledge of the prevalence of structural variation in populations and its impact on health and evolution over the past two decades. Numerous algorithms and approaches have been designed and employed to detect structural variants from short-read sequencing (SRS) data (reviewed in Kosugi et al., 2019). While many of them represent an improvement in some specific aspect, they all suffer from three basic problems, associated with technical limitations inherent to short reads. First, no single algorithm can detect SVs of all types and sizes. As shown by an exhaustive study that compared the performance of 69 existing algorithms for SV detection from WGS data, most algorithms perform best for particular SV types and, in some cases, for particular size ranges (Kosugi et al., 2019). Even when the same approach and the same algorithm is used, substantial differences may exist between samples that are not due to biological differences. For example, in the read-depth approach, lower coverage will lead to fewer SVs being identified, the power to detect smaller events will be compromised and neighboring SVs may collapse into single calls due to diminished resolution (Pezer et al., 2015). These problems make comparisons between studies difficult, as each approach applied to the same biological sample will result in a different set of SVs.

Second, the true positive rate of SRS-based methods is generally low while the false positive rates can be as high as 90%; again, both are heavily dependent on the size and type of SVs (Mahmoud et al., 2019). Differences in the processing of samples and data before SV calling can also strongly affect the accuracy of the final call set. For example, Khayat et al. (2021) found that sequencing centers and especially read mapping methods contribute significantly to variability between call sets. In particular, their results suggest that one-fifth of all calls represent false positives that are solely contributed by the mapper. These problems have major consequences on reproducibility and can greatly affect the interpretation between studies.

Third, methods based on SRS are unable to (accurately and reliably) identify SVs in repetitive genomic regions, stemming from the uncertainty of the true origin of reads that can be equally well mapped to multiple genomic positions. As a consequence, these problematic, repetitive regions are often omitted in genomic analyses. However, recent analyses based on long-read sequencing (LRS) technologies suggest that these regions may be the greatest source of variation. In human genomes, up to 90% of SVs (mostly smaller than 1 kbp) detected from LRS data were unknown from previous SRS-based analyses (Chaisson et al., 2015; Huddleston et al., 2017; Audano et al., 2019; Ebert et al., 2021; Quan et al., 2021). This means that SRS-based methods are blind to the vast majority of variation. This problem is particularly relevant in analyses of genomes with high repetitive DNA content such as in many plant species.

Despite its power to detect variation that is inaccessible to SRS, LRS has several drawbacks which directly limit its use in large population studies: it is more expensive, requires more input DNA, and has lower sample throughput than SRS (Ho et al., 2020). Consequently, not many population genomic studies based on long reads have emerged so far (Audano et al., 2019; Weissensteiner et al., 2020; Beyter et al., 2021; Quan et al., 2021; Yan et al., 2021; Rech et al., 2022). Majority of these studies employ a hybrid strategy which involves sequencing a smaller number of genomes by using long reads while the remaining samples are sequenced with short-read technology (Ho et al., 2020; De Coster et al., 2021; Quan et al., 2022). Structural variants identified by LRS in representative genomes can then be genotyped from SRS data in all other samples. This approach combines the advantages of both read-sequencing technologies: the power of LRS to discover multiple types and a wider size range of SVs (Quan et al., 2022), and the generally high genotyping precision of SRS-based algorithms (Kosugi et al., 2019). Even so, not all SVs that are detected from long reads can be accurately genotyped from short-read data, and as much as half remain invisible to it (Huddleston et al., 2017; Chakraborty et al., 2019; Ebert et al., 2021). Furthermore, LRS produces reads that are still insufficiently long to resolve all SVs. For instance, detection algorithms based on long reads that consider information on soft clipped reads and intra-read discordance are much worse at discovering CNVs larger than >100 kbp than are algorithms based on the read-depth approach from short reads (Kosugi et al., 2019). Hence, despite the advances made related to improved identification of smaller SVs by long reads, much of the most complex genomic regions remains inaccessible. Optical mapping is a technology of choice for resolving such regions as it generates molecules that can be over 1 Mb long and can therefore bridge larger repetitive regions (Ho et al., 2020). It has been successfully applied in some population studies which resolved previously undetected large SVs and identified novel genome content not found in the reference genome sequence (Levy-Sakin et al., 2019; Weissensteiner et al., 2020). However, optical mapping has several weak points, such as a high error rate, a lack of information on the actual sequence underlying the molecules, and the inherent inability to determine precise SV breakpoints. The widespread use of optical mapping is further hindered by lower throughput and the lack of alternative and publicly available tools for SV detection (see Li et al., 2017; Raeisi Dehkordi et al., 2021). Another promising technology that has the potential to detect large SVs and those in repetitive regions is high-throughput chromosome conformation capture (Hi-C). Hi-C is typically used for studying 3D genome interactions, and although several tools have been developed for SV discovery from Hi-C data, these are specifically designed for human genomes and are limited to the detection of SVs larger than 1 Mbp. Most recently, a framework named EagleC was developed that has the power to detect events down to 1 kb in any species genome, providing sufficient coverage (Wang et al., 2022b). This tool illustrates the potential of Hi-C application in SV discovery from large sample sets, and further developments in this direction will enable widespread and more comprehensive population-scale studies of SVs by use of Hi-C technology.

### Towards more representative reference genome

In population studies, structural variants are most commonly detected from sequencing data by aligning reads to the reference genome sequence and identifying patterns of discordance in alignment. If the reference genome is contiguous, an average read depth of 10x is considered sufficient for population-scale comparisons (Collins et al., 2020). However, reference genomes assembled at the chromosome level are rarely available, which hampers studies in the majority of species. Even human genomes seem to contain large regions not present in the reference genome, as shown by studies based on optical mapping and long reads (Audano et al., 2019; Levy-Sakin et al., 2019; Ebert et al., 2021). They are not merely repetitive and non-functional, but also encompass genes and regulatory elements. These studies question the completeness and the representativeness of the human reference genome. The latest version of the human reference assembly, T2T-CHM13, succeeded in closing all gaps found in the previous GRCh38 assembly and indeed represents the first completely sequenced genome (Nurk et al., 2022). However, similar to the GRCh38, in which the majority of sequence originates from a single individual (Ballouz et al., 2019), T2T-CHM13 represents only one haplotype, and while it improves analysis of human genetic variation to some extent (Aganezov et al., 2022), it cannot fully capture the genetic diversity among populations. Approaches to remove reference bias have started to emerge, to improve accuracy in population-scale SV analyses. In 2019, Sherman et al. (2019), sequenced 910 individuals of African descent and used all unaligned reads to assemble contigs *de novo*. These collectively constituted 300 million base pairs of sequences that were missing from the reference genome and illustrated that a single reference genome is suboptimal for population-based studies. Instead, the creation of a comprehensive pan-genome was proposed, based on all distinct human populations that would much better capture all the DNA present in humans. In 2019, the Human Pangenome Project was initiated, funded by the US National Human Genome Research Institute (NHGRI), with a goal to provide a more accurate and diverse representation of global genomic variation through the creation of a more sophisticated human reference genome (Wang. et al., 2022a; Khamsi, 2022).

Pangenomes are superior to single reference genomes because they combine genomes from multiple individuals and thus better incorporate genomic polymorphism within a population, and they are becoming increasingly used for SV studies in humans and other species (Beyter et al., 2021; Ebert et al., 2021; Qin et al., 2021; Yan et al., 2021; Zhou et al., 2022; for a list of studies based on plant pan-genomes see Yuan et al., 2021). Instead of being represented as a linear sequence, pangenomes are constructed as graphs to which sequencing reads are aligned (De Coster et al., 2021; Quan et al., 2022), enabling reliable genotyping of SVs by short reads in thousands of samples, which facilitates large population studies. However, approaches for graph-based genotyping are in their infancy, and tools for more efficient construction of complex graphs and alignment of reads to graphs are still under development (Quan et al., 2022).

### The power of haplotype-resolved genomes

One of the major obstacles to a deeper understanding of SVs is the inability to accurately determine discrete SV alleles as it hinders evolutionary and population genetic studies of SVs, including analyses of allele frequency, estimations of the rates of recurrent mutation and incorporation of SVs in genome-wide association studies (Ebert et al., 2021; Saitou et al., 2022). This limitation can be overcome by resolving haplotypes. Studies that analyze haplotype-resolved genomes readily identify a substantial number of previously undetected SVs and additional genomic content not present in the reference genome (Huddleston et al., 2017; Wong et al., 2018; Chaisson et al., 2019; Levy-Sakin et al., 2019; Almarri et al., 2020; Ebert et al., 2021; Hämälä et al., 2021). Huddleston et al. (2017) sequenced genomes of two hydatiform moles - which are haploid; when they merged the two haploid genomes *in silico* to create an artificial diploid genome, over half of the heterozygous SVs were no longer detected from long-read sequencing data. This showed that the majority of SVs are not detectable unless the haplotype structure of the genomes is known and illustrates the importance of haploid resolution for the sensitivity of SV detection. However, determining the physical haplotype structure of genomes is yet not widely affordable and the haplotype-phasing methods are still immature, preventing their wider application in population-scale studies.

## Variant interpretation

Over the past two decades, a sheer abundance of studies has demonstrated that structural variants are by far the most dominant form of genetic variation. While our ability to detect SVs has increased tremendously, for the largest part we are still unable to explain the functional consequences (Ho et al., 2020; Yan et al., 2021). For example, in the majority of population-scale studies, evidence on the adaptive role of SVs is inferred from associations between SV frequency and environmental/behavioral traits, however, rare studies provide evidence based on phenotypic assays, such as gene expression and protein level, or fitness (Perry et al., 2007; Maron et al., 2013; Ishikawa et al., 2019; Zhao et al., 2020). Experimental evolution provides a powerful means to study adaptation, yet it is limited to species with short generation times such as single-cell organisms. In more complex, multicellular organisms, it was proposed that integrating SVs with layered biological data is crucial for a more complete understanding of the impact of SVs (Ho et al., 2020). These may include but are not limited to analyses of transcriptome, epigenome, proteome, and 3D chromatin structure.

## Concluding remarks

Recent years have shown that genome plasticity is even larger than it was anticipated more than 10 years ago. Long-read sequencing technologies have enabled the discovery of a wealth of structural variation in previously inaccessible genomic regions and continuous efforts provide increasing evidence that SVs play important roles in population divergence, local adaptation, and speciation. However, there is currently no approach that would allow simultaneous detection of all SVs, and even methods based on long reads fail in complex genomic regions such as long tandemly repetitive sequences and segmental duplications. Therefore, systemic assessments of SVs’ contribution to evolution are primarily hindered by the high cost of analyzing a large number of individuals to enable population-scale studies, and by the necessity to employ multiple available technologies, in order to capture all types of SVs and achieve greater resolution of SV detection. Without a such comprehensive approach, the investigations are limited to SVs of a particular size range or types. Pangenome assemblies provide a route to avoid costly sequencing by long reads and enable genotyping from short reads mapped on a reference genome that is derived from several individuals, representative of multiple populations. Investment in efforts to construct pangenomes in a multitude of species will enable more reliable and comprehensive SV detection and genotyping on a larger scale. The number of detected SVs is expected to increase further with the improvement of haplotype-phasing methods, and the wider application of such methods is expected to greatly advance our understanding of the impact of SVs on evolution.

Box 1 **SVs in numbers**

-92,934 common structural variant regions in human populations; according to the NCBI Curated Common Structural Variants dataset (dbVar study accession nstd186; Lappalainen et al., 2013)
-27,662 SVs detected per person, including STRs and other highly repetitive elements (Chaisson et al., 2019)
-16 Mbp—The average amount of structural variation per person (Ebert et al., 2021)
-3–15X—More base pairs are affected by SVs than by SNVs (Pang et al., 2010; Huddleston et al., 2017; Hämälä et al., 2021)
-3–10X—Higher inter-individual genomic difference at SVs than at SNVs (Pang et al., 2010; Sudmant et al., 2015b)
-4.8%–9.5% of the human genome is affected by CNVs (Zarrei et al., 2015)
-0.29—Number of *de novo* SVs per generation (in regions of the genome accessible to short-read sequencing) or one new SV every two to eight live births (Collins et al., 2020; Belyeu et al., 2021)
-6.8%—the largest estimated proportion of eQTLs caused by SVs (Chiang et al., 2017)
